# Molecular Evolution of Human Parainfluenza Virus Type 2 Based on Hemagglutinin-Neuraminidase Gene

**DOI:** 10.1128/spectrum.04537-22

**Published:** 2023-04-11

**Authors:** Yi Feng, Zhen Zhu, Jin Xu, Liwei Sun, Hui Zhang, Hongmei Xu, Feng Zhang, Wenyang Wang, Guangyue Han, Jie Jiang, Ying Liu, Shanshan Zhou, Yan Zhang, Yixin Ji, Naiying Mao, Wenbo Xu

**Affiliations:** a National Health Commission (NHC) Key Laboratory of Medical Virology and Viral Diseases, National Institute for Viral Disease Control and Prevention, Chinese Center for Disease Control and Prevention, Beijing, China; b Henan Provincial Center for Disease Control and Prevention, Zhengzhou, China; c Changchun Children's Hospital, Changchun, China; d Gansu Provincial Center for Disease Control and Prevention, Lanzhou, China; e Children's Hospital of Chongqing Medical University, Chongqing, China; f Qingdao Center for Disease Control and Prevention, Qingdao, China; g Department of Immunology, School of Medicine, Anhui University of Science and Technology, Huainan, China; h Hebei Provincial Center for Disease Control and Prevention, Shijiazhuang, China; U.S. Food and Drug Administration

**Keywords:** Bayesian analysis, human parainfluenza virus type 2, genotyping, molecular evolution

## Abstract

To understand the molecular evolution of human parainfluenza virus type 2 (HPIV2), 21 Hemagglutinin-Neuraminidase (HN) gene sequences covering seven Chinese provinces in 2011 and 2017 to 2021 were combined with 90 published HN sequences worldwide for phylogenetic analysis. The result showed that global HPIV2 could be classified into two distinct clusters (I and II), five lineages (IA to IIE), and four sublineages (IB1 and 2, and IIE1 and 2). The minimum genetic distances between different clusters and lineages were 0.049 and 0.014, respectively. In the last decade, one lineage (IID) and three sublineages (IB1, IB2, and IIE1) have been cocirculating in China, with the sublineages IB2 and IIE1 dominating, while sublineages IB1 and IIE1 are dominant globally. In addition, the spread of HPIV2 had relative spatial clustering, and sublineage IB2 has only been detected in China thus far. The overall evolution rate of HPIV2 was relatively low, on the order of 10^−4^ substitutions/site/year, except for sublineage IB2 at 10^−3^ substitutions/site/year. Furthermore, human–animal transmission was observed, suggesting that the HPIV2 might have jumped out of animal reservoirs in approximately 1922, the predicted time of a common ancestor. The entire HN protein was under purifying/negative selection, and the specific amino acid changes and two novel N-glycosylation sites (N316 and N517) in sublineages IB1, IB2, and IIE1 were mostly located in the globular head region of the HN protein. In this study, preliminary evolutionary characteristics of HPIV2 based on the HN gene were obtained, increasing the recognition of the evolution and adaptation of HPIV2.

**IMPORTANCE** The phylogenetic analysis showed that global HPIV2 could be classified into two distinct clusters (I and II) and five lineages (IA to IIE) with at least 0.049 and 0.014 genetic distances between clusters and lineages, respectively. Furthermore, lineages IB and IIE could be further divided into two sublineages (IB1-2 and IIE1-2). All China sequences belong to one lineage and three sublineages (IB1, IB2, IID, and IIE1), among which sublineages IB2 and IIE1 are predominant and cocirculating in China, while sublineages IB1 and IIE1 are dominant globally. The overall evolution rate of HPIV2 is on the order of 10^−4^ substitutions/site/year, with the highest rate of 2.18 × 10^−3^ for sublineage IB2. The entire HN protein is under purifying/negative selection, and the specific amino acid substitutions and two novel N-glycosylation sites (N316 and N517) in sublineages IB1, IB2, and IIE1 are mostly located in the globular head region of the HN protein.

## INTRODUCTION

Human parainfluenza virus (HPIV) is a member of the family Paramyxoviridae, and it has a single-stranded and negative-sense RNA genome. HIPVs are composed of four distinct serotypes, including HPIV1, HPIV2, HPIV3, and HPIV4, among which HPIV2 belongs to the genus *Rubulavirus* within the Orthoparamyxovirinae subfamily and can cause a wide spectrum of respiratory illnesses from mild upper respiratory infections to severe bronchitis and pneumonia in children, immunocompromised patients, and the elderly (1 to 5). A systemic review in 2021 estimated that infection with HPIV1–4 caused approximately 13% of acute lower respiratory infection (ALRI) cases, 4 to 14% of ALRI hospital admissions, and 4% of ALRI mortality worldwide among children younger than 5 years (5). Generally, HPIV2 prevalence was ranked after HPIV3 and HPIV1, accounting for 2.6 to 14% of HPIV infections, depending on the case definition, detection method, and duration of the studies (6 to 9). However, due to the lack of systematic surveillance, the disease burden of HPIV2 in China remains unclear.

The genome of HPIV2 is approximately 15,600 bp in length, and it encodes eight functional proteins. Among them, the hemagglutinin-neuraminidase (HN) protein, which is mainly involved in the binding process of the virus to the sialic acid receptor on the host cell surface, shows the largest antigenic and genetic differences (10 to 13). Therefore, the HN gene has been widely used in molecular epidemiological studies of HPIVs (14 to 20). In 2008, based on the HN gene, Terrier et al. classified HPIV2 into four evolutionary branches, which were prevalent in Japan, the United States, France, and the United Kingdom before 2001 (21). Subsequently, several studies further classified HPIV2 into four genotypes (G1 to G4) and five subgenotypes (G1a to c, G4a and b) based on the HN gene (22 to 24). In China, due to the few available HN sequences, the genotype distribution and molecular evolutionary patterns of HPIV2 have not been fully clarified.

With the development of sequencing technology and the emphasis on the disease burden of HPIV2 in the context of the severe acute respiratory syndrome coronavirus 2 (SARS-CoV-2) epidemic, more HN sequences of HPIV2 have been accumulated globally. Therefore, we carried out genetic characteristics and molecular evolution studies on HPIV2 circulating in China and even worldwide, aiming to enrich the research data on HPIV2, reveal the latest evolutionary characteristics of HPIV2, and provide a basis for disease surveillance, vaccine development, antiviral research, and prevention and control.

## RESULTS

### Case information.

In 2011 and 2017 to 2021, a total of 21 HN sequences of HPIV2 were obtained from acute respiratory infection (ARI) pediatric cases in seven provinces of China (Henan: 9; Jilin: 6; Gansu: 2; Chongqing: 1; Shandong: 1; Hebei: 1; Anhui: 1). The sequences of HPIV2 were not available for the period 2012 to 2016 due to the lack of the active surveillance of HPIV in China in this study. The age of the 21 cases ranged from 1 to 12 years, with a median age of 3 years. Except for 2 cases without clinical information, 13 of 19 cases (68.4%) presented as lower respiratory tract infections, and the main clinical diagnoses were acute bronchitis (31.6%), bronchopneumonia (21.1%), and pneumonia (15.8%). In addition, 9 (42.9%) of the 21 cases were coinfected with other respiratory viruses, including 6, 1, 1, and 1 cases of duel, triple, quadruple, and quintuple coinfection, respectively. Multiple viral pathogens, including human respiratory syncytial virus (HRSV), human adenovirus (HAdV), and human rhinovirus (HRV), were the main pathogens for coinfections, accounting for 33.3%, respectively. The major clinical manifestations of the coinfections were bronchopneumonia (44.4%) (Table S5 in the supplemental material).

### Phylogeny of HPIV2 worldwide.

**FIG 1:**
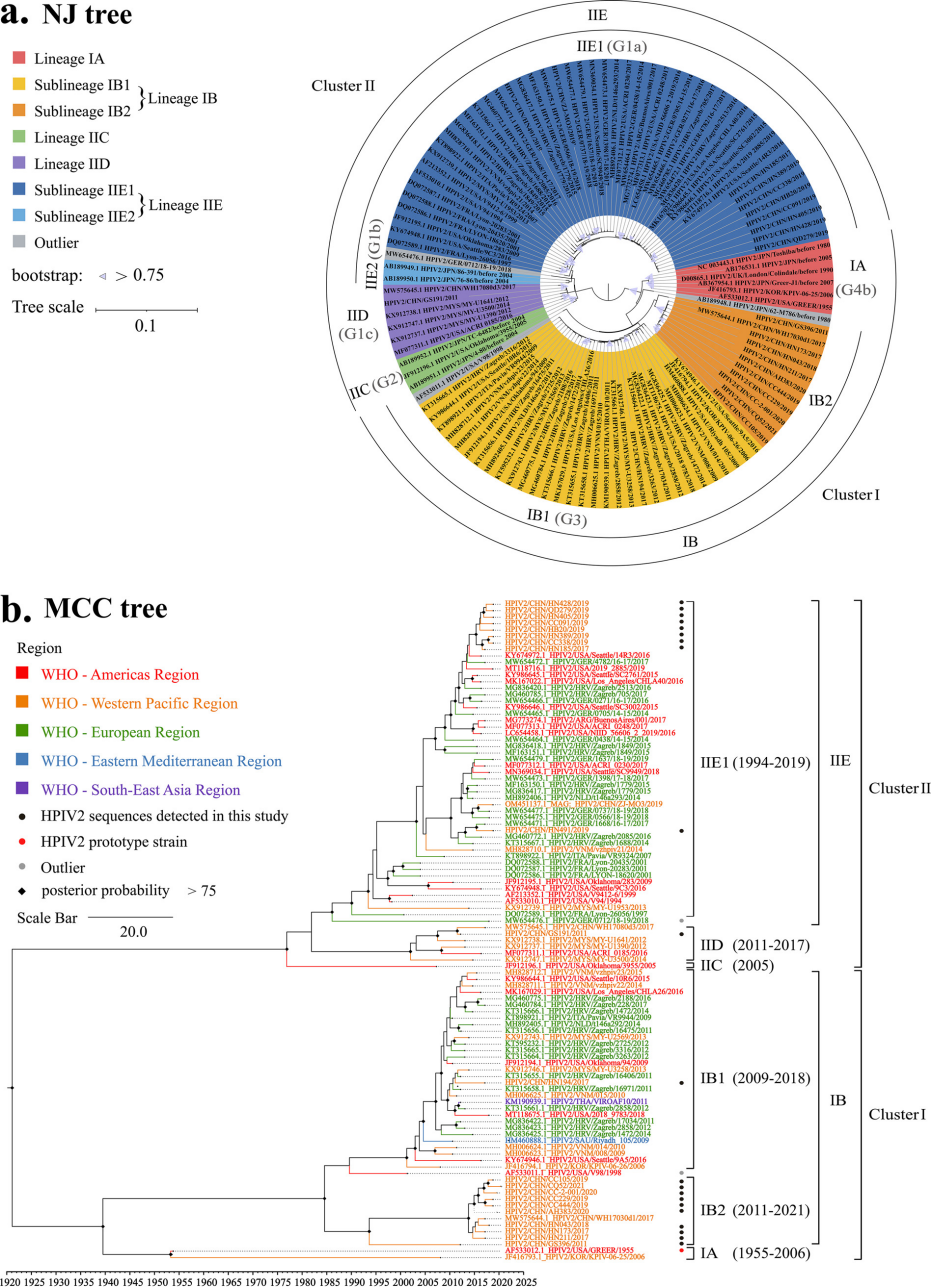
Neighbor-Joining (NJ) phylogenetic tree (a) and Bayesian phylogenetic tree (b) based on the HPIV2 HN gene. The previous genotype is represented in parentheses. The names of the strains include the GenBank number, serotype, country of isolation, name, and age of the isolation. The country abbreviations ARG, CHN, FRA, GER, HRV, ITA, JPN, KOR, MYS, NLD, SAU, THA, UK, USA, and VNM in the trees represent Argentina, China, France, Germany, Croatia, Italy, Japan, South Korea, Malaysia, the Netherlands, Saudi Arabia, Thailand, the United Kingdom, the United States, and Vietnam, respectively. The abbreviations AH, GS, HB, HN, CQ, and ZJ in the trees represent Anhui, Gansu, Hebei, Henan, Chongqing, and Zhejiang provinces in China, respectively. The abbreviations CC, WH, and QD in the trees refer to Changchun city of Jilin province, Wuhan city of Hubei province, and Qingdao city of Shandong province, respectively.

**FIG 2:**
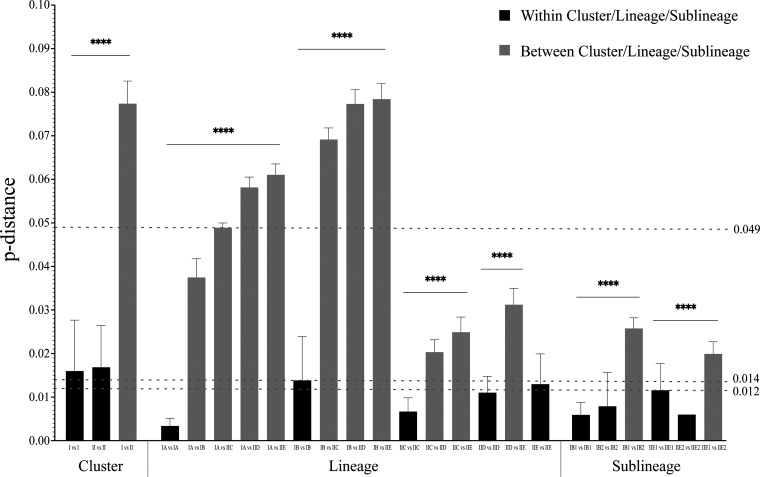
Pairwise comparison of genetic distance between and within groups of HPIV2. ****, *P* < 0.001, showing the statistically significant difference. The abbreviations I, II, IA, IB, IIC, IID, E, IB1, IB2, IIE1, and IIE2 in the figure referred to cluster I, cluster II, lineage IA, lineage IB, lineage IIC, lineage IID, lineage E, sublineage IB1, sublineage IB2, sublineage IIE1, and sublineage IIE2, respectively.

According to the phylogenetic tree, the 111 global representative HN sequences of HPIV2 could be divided into two major clusters (cluster I and cluster II) and five distinct evolutionary lineages (IA, IB, IIC, IID, IIE); among them, lineages IA and IB belonged to cluster I, and IIC, IID, and IIE belonged to cluster II (Fig. 1). The genetic distance between cluster I and cluster II was 0.075 (0.049 to 0.091), and the genetic distances within cluster I and cluster II were 0.020 (0.000 to 0.048) and 0.017 (0.001 to 0.039), respectively. The genetic distances between and within the different lineages were 0.038 and 0.003 to 0.014 in cluster I and 0.020 to 0.031 and 0.007 to 0.013 in cluster II. Furthermore, two sublineages could be identified for both lineages IB (IB1 and IB2) and IIE (IIE1 and IIE2), and the genetic distances between and within the sublineages were 0.026 and 0.006 to 0.008 in lineage IB and 0.020 and 0.006 to 0.012 in lineage IIE, respectively (Tables S6 to S8). The differences in the division for clusters (0.049), lineages (0.014), and sublineages (0.012) were statistically significant using nonparametric tests for pairwise p-distance (Fig. 2). It should be noted that the genetic distances between three sequences (strain 62-M786 from Japan before 1980: AB189948; strain V98 from the Unite States in 1998: AF533011; and strain 0712 from Germany in 2018: MW654476) and other sequences were 0.019 to 0.064, 0.014 to 0.026, and 0.026 to 0.034, respectively, which were higher than the threshold values for lineage (0.014) and sublineage (0.012) division in this study (Tables S6 to S8). Therefore, these three sequences were considered outliers because no similar sequences have been detected so far.

Lineage IA, IIC, and IID strains were sporadically detected around the world before 2017. Among them, lineage IA refers to the previous subgenotype G4b containing the prototype strain of HPIV2 from America in 1955 (strain GREER: AF533012) and five HN sequences from the United Kingdom, Japan, and South Korea. Lineage IIC refers to genotype G2 containing HN sequences from Japan before 2004 and the United States in 2005. Lineage IID corresponds to the previous subgenotype G1c containing HN sequences detected in Malaysia, the United States, and China between 2012 and 2017.

Compared with the above three lineages, lineages IB and IIE showed a spatial clustering trend. Lineage IB corresponded with the published genotype G3 that was first identified in 1998, and it continued to circulate in 10 countries (Croatia, Italy, South Korea, Malaysia, Thailand, Netherlands, Saudi Arabia, the United States, Vietnam, and China) during 1998 to 2021. Among the two sublineages of lineage IB, IB1 was most prevalent during 2006 to 2018, while IB2 showed relatively geographic restrictions and was detected only in China in 2011 and 2017 to 2021.

Lineage IIE corresponded to the combination of the previous subgenotypes G1a and G1b and was first detected in 1994 and then circulated in 11 countries (Argentina, France, Germany, Croatia, Italy, Japan, Malaysia, the Netherlands, the United States, Vietnam, and China) during 1994 to 2019. Of the two sublineages, IIE1 was the most widely distributed during 1994 to 2019, while IIE2 was only sporadically detected in Japan before 2004 (Table S9). Most of the sequences (72.07%) in the data set belonged to sublineages IB1 and IIE1, indicating that these two sublineages were the most predominant viruses of HPIV2 worldwide. In addition, both sublineages IB1 and IIE1 formed a typical ladder-like topology in the phylogenetic tree. Although two distinct branches within sublineage IIE1 were found with strong support of the bootstrap value (>75%), this sublineage was not further classified due to the inclusion of 20% scattered sequences.

### Phylogenetic analysis of HPIV2 in China.

Together with three Chinese HN sequences of HPIV2 downloaded from GenBank, a total of 24 HN sequences covering 9 provinces (Gansu, Anhui, Hebei, Henan, Jilin, Chongqing, Shandong, Hubei, and Zhejiang) were used in this study. All Chinese HN sequences of HPIV2 shared 91.2 to 99.9% nucleotide identity and 93.5 to 100% amino acid identity. Based on the phylogenetic analysis, 24 sequences in 2011 and 2017 to 2021 could be classified into four evolutionary lineage/sublineages (*IID*, IB1, IB2, and IIE1), which were relatively clustered within the lineage/sublineages.

Except for lineage IID and sublineage IB1, which were sporadically detected in China, most sequences (79.2%) belonged to sublineages IB2 (11 from 6 provinces during 2011 to 2021) and IIE1 (10 from 5 provinces during 2017 to 2019), respectively. In addition, Chinese HPIV2 sequences formed an independent evolutionary branch in both sublineages. The nucleotide and amino acid identities of Chinese sublineages IB2 and IIE1 were 97.2 to 99.8% and 96.8 to 100%; 98.3 to 99.9% and 99.1 to 100%, respectively. The above results indicated that sublineages IB2 and IIE1 have been cocirculating in China (Fig. 1).

### Evolutionary rate, the most recent common ancestor, and effective population size.

The Bayesian analysis showed that the mean evolutionary rate of global HPIV2 HN sequences was estimated at approximately 4.53 × 10^−4^ substitutions/site/year (95% highest posterior density [HPD]: 3.80 × 10^−4^ to 5.27 × 10^−4^) and probably emerged in 1922 (95% HPD:1908 to 1935). For clusters, clusters I and II could be traced back to 1934 (95% HPD:1894 to 1955) and 1985 (95% HPD:1980 to 1990), with estimated evolutionary rate at 4.46 × 10^−4^ substitutions/site/year (95% HPD: 2.77 × 10^−4^ to 6.12 × 10^−4^) and 3.94 × 10^−4^ substitutions/site/year (95% HPD: 3.38 × 10^−4^ to 4.59 × 10^−4^), respectively. The mean evolutionary rates of lineages IB and IIE were very similar, at 5.44 × 10^−4^ substitutions/site/year (95% HPD: 3.74 × 10^−4^ to 7.25 × 10^−4^) and 6.06 × 10^−4^ substitutions/site/year (95% HPD: 4.75 × 10^−4^ to 7.44 × 10^−4^), respectively. The time to the most recent common ancestor (tMRCA) of lineages IB and IIE was estimated in 1988 (95% HPD:1979 to 1996) and 1987 (95% HPD:1981 to 1991), respectively. The sublineage IB2 found only in China showed the highest evolutionary rate of 2.18 × 10^−3^ (95% HPD: 8.29 × 10^−4^ to 3.36 × 10^−3^), and it could be traced back to 2009 (Table 1). In comparison, sublineages IB1 and IIE1 showed relatively lower rates of 3.97 × 10^−4^ (95% HPD: 1.77 × 10^−4^ to 6.17 × 10^−4^) for IB1 and 5.81 × 10^−4^ (4.37 × 10^−4^ to 7.30 × 10^−4^) for IIE1 (Table 1).

**TABLE 1:** The time to the most recent common ancestor (tMRCA) and evolutionary rate of HPIV2

Type	No. of strains	Period	tMRCA (95% HPD)	Evolutionary rate (95% HPD)
ALL HPIV2	102	1955 to 2021	1922 (1908 to 1935)	4.53 × 10^−4^ (3.80 × 10^−4^ to 5.27 × 10^−4^)
Cluster I	45	1955 to 2021	1934 (1894 to 1955)	4.46 × 10^−4^ (2.77 × 10^−4^ to 6.12 × 10^−4^)
Cluster II	57	1994 to 2019	1985 (1980 to 1990)	3.94 × 10^−4^ (3.38 × 10^−4^ to 4.59 × 10^−4^)
Lineage IB	43	1998 to 2021	1988 (1979 to 1996)	5.44 × 10^−4^ (3.74 × 10^−4^ to 7.25 × 10^−4^)
Sublineage IB1	31	2009 to 2018	1996 (1987 to 2004)	3.97 × 10^−4^ (1.77 × 10^−4^ to 6.17 × 10^−4^)
Sublineage IB2	11	2011 to 2021	2009 (2003 to 2011)	2.18 × 10^−3^ (8.29 × 10^−4^ to 3.36 × 10^−3^)
Lineage IIE	50	1994 to 2019	1987 (1981 to 1991)	6.06 × 10^−4^ (4.75 × 10^−4^ to 7.44 × 10^−4^)
Sublineage IIE1	49	1994 to 2019	1988 (1983 to 1992)	5.81 × 10^−4^ (4.37 × 10^−4^ to 7.30 × 10^−4^)

**FIG 3:**
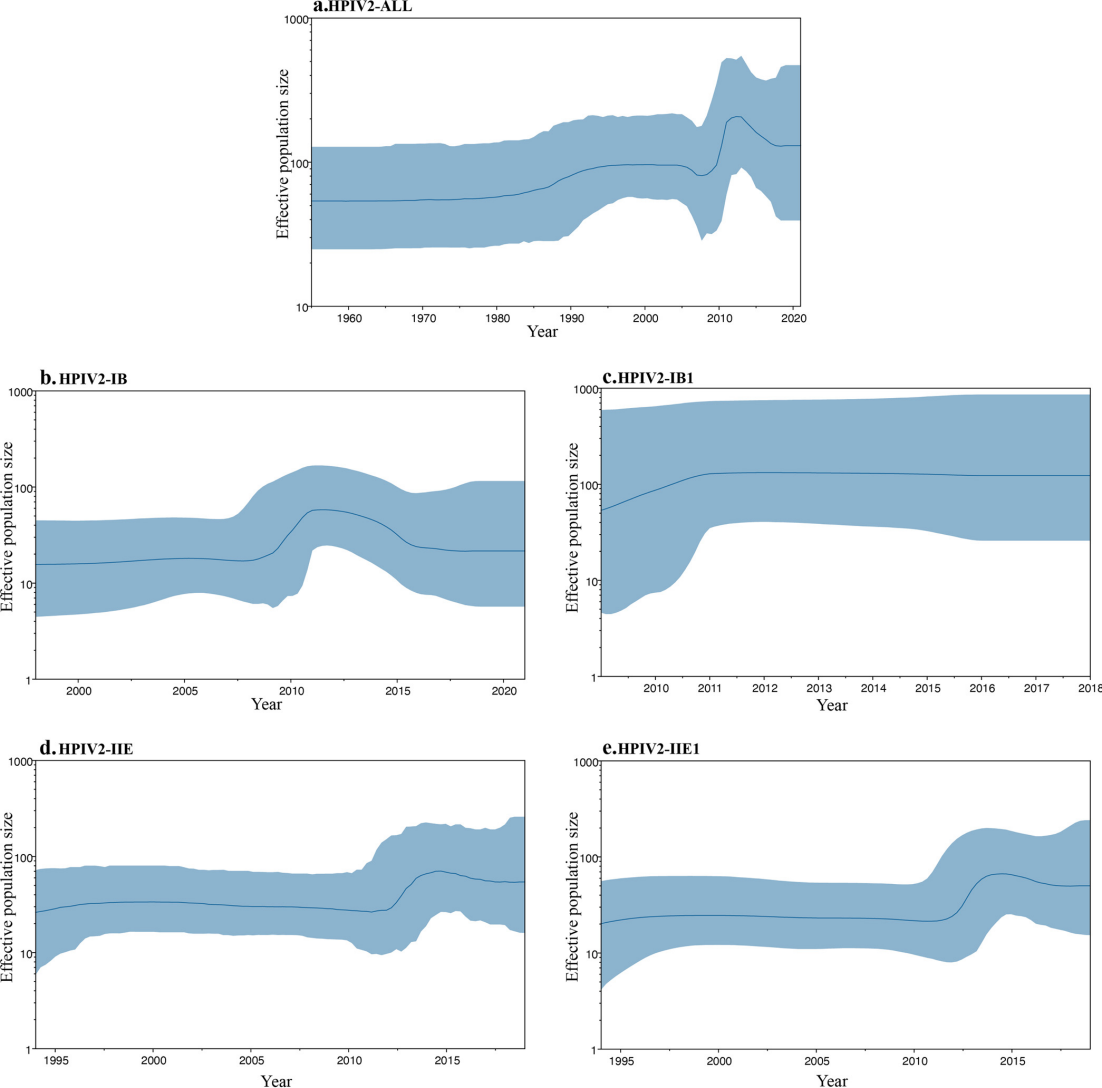
Bayesian skyline plot of HPIV2 based on the HN gene sequences. (a) Bayesian skyline plot construed with all 111 sequences; (b) Bayesian skyline plot construed with 43 sequences of lineage IB; (c) Bayesian skyline plot construed with 31 sequences of sublineage IB1; (d) Bayesian skyline plot construed with 52 sequences of lineage IIE; (e) Bayesian skyline plot constructed with 49 sequences of sublineage IIE1.

The genetic diversity of HPIV2 lineages based on Bayesian skyline models was evaluated. The Bayesian skyline plot (BSP) revealed that the global HPIV2 effective population experienced multiple shifts in size. The first expansion of the HPIV2 effective population size occurred around 1980 to 2005, followed by a second rapid expansion in approximately 2010 to 2015. Meanwhile, the expansions of lineages IB and IIE were observed in almost the same period as the second expansion of HPIV2, which might be due to the expansion of sublineages IB1 and IIE1 (Fig. 3).

### Amino acid substitutions.

Amino acid substitutions were identified for each lineage of HPIV2 based on the HN gene. All HPIV2 sequences encoded 571 amino acids without insertions or deletions. The HPIV2 HN protein is composed of four functional domains: a cytoplasmic tail (aa [amino acids] 1 to 18), a transmembrane region (aa 19 to 37), a stalk region (aa 38 to 119), and a globular head region (aa 120 to 571) (25). Compared with the prototype strain, lineage IB shared 14 amino acid substitutions (2 in the stalk region: D54N and I67V; 12 in the globular head region: N164H, I175S, K316N, K323E, K341N, S351G, V367I, H376Q, A416S, R497K, S513N, and A514S), among which seven amino acid changes (D54N, I67V, N164H, I175S, S351G, V367I, and H376Q) were unique. Similarly, lineage IIE shared 14 amino acid changes in the globular head region (K139E, T195A, A201S, P319T, K323E, K341N, A348I, A378E, A416S, T480M, Q482R, S513N, I570M, and P571L), among which two amino acid changes (A378E and I570M) were unique (Tables S10 to S13). Additionally, sublineage-specific amino acid changes in the HN protein were observed; for example, sublineages IB1 and IB2 had their own unique substitutions in the globular head region (IB1: N360Y; IB2: P319S) (Table S10).

Compared with the sequences of the earliest reported strains of lineages IB and IIE, 10 amino acid substitutions were observed in more than 20% of lineage IB sequences (2 in the stalk region: A48V and T117S; 8 in the globular head region: K139N, E254D, V276T, N322K, A348T, N360Y, D381N and D381S), and six substitutions were found in more than 20% of lineage IIE sequences (1 in the stalk region: E57D; 5 in the globular head region: V137A, M186I, E254D, S351G, and D476N). For sublineages, IB1 and IB2 shared one substitution each at N360Y and A48V, respectively, and IIE2 shared four substitutions at N54D, V87I, H164N, and M186I (Tables S14 and S15).

### N-glycosylation site prediction and selection pressure analysis.

The analysis of potential N-glycosylation sites showed that four N-glycosylation sites (N6, N115, N142, and N272) were highly conserved in all HN sequences of HPIV2 strains, except for two sequences from Japan (strain 62-M786: AB189948; strain 86-391: AB189949), which lost one N-glycosylation site at positions N142 and N115, respectively. Additionally, all sequences except lineage IA shared two novel N-glycosylation sites (N316 and N517), with the exception of five sequences. Among the five sequences, two from Japan (outlier: strain 62-M786, AB189948; sublineage IIE2: strains 86-391, AB189949) and one from the United States (sublineage IIE1: strain Los_Angeles CHLA40, MK167022) lost the N-glycosylation site at position N316; one from Malaysia (lineage IID: strain MY-U3500, KX912747) and one from Germany (sublineage IIE1: strain 1637 18-19, MW654479) missed the N-glycosylation site at N517 (Table S16).

Selection pressure analysis of HN proteins revealed that lineage IID and sublineages IB1, IB2, and IIE1 had 1, 12, 4, and 24 negative selection sites, respectively, with evolutionary pressure values of ω < 1. In contrast, no positive selection site was found because five predicted sites were only supported by one algorithm (Table 2; Tables S17 to S21).

**TABLE 2:** Selection pressure analysis of lineage IID and sublineages IB1, IB2, and IIE1 based on HN protein^*a*^

Type	MEME		SLAC		FEL		FUBAR	
	PSS	NSS	PSS	NSS	PSS	NSS	PSS	NSS
Sublineage IB1	0	0	0	3	0	8	1	12
Sublineage IB2	1	0	0	0	0	3	0	1
Lineage IID	0	0	0	0	0	1	1	0
Sublineage IIE1	0	0	0	6	0	21	2	24

a PSS, presented positive selection site; NSS, presented negative selection site.

## DISCUSSION

Since the Corona Virus Disease-19 (COVID-19) outbreak occurred in late 2019, SARS-CoV-2 transmission and the mitigation measures against the pandemic had an impact on the epidemiology of almost all respiratory viruses. Compared with prepandemic years, the global prevalence of HPIV tended to be increased in some countries (25 to 27). Therefore, it is very necessary to carry out genetic variation and molecular evolution analysis of HPIV2 to understand its global transmission pattern, and the accumulation of HN sequences worldwide offers this possibility. In this study, based on phylogenetic analysis, global HPV2 was reclassified into two clusters (I and II) and five evolutionary lineages (IA to IIE), and lineages IB and IIE could be further divided into two sublineages (IB1 and 2; and IIE1 and 2). The minimum genetic distance between clusters and lineages was 0.049 and 0.014, which could be proposed as the threshold for cluster and lineage division of HPIV2. All China sequences in 2011 and 2017 to 2021 showed genetic diversity and belonged to one lineage (IID) and three sublineages (IB1, IB2, IIE1), among which sublineages IB2 and IIE1 were predominant and cocirculating in China, while sublineages IB1 and IIE1 were dominant globally. The differences observed in the above dominant epidemic lineages in this study might be related to limited genetic data and sampling bias. So far, only sublineage IB2 was detected during the COVID-19 pandemic in China, and whether the mitigation measures against the pandemic had an impact on the transmission pattern of HPIV requires more data to be clarified.

Additionally, the results of this study showed that HPIV2 coinfection was frequently observed, but no correlation was found with clinical symptoms and lineage/sublineage distribution. The most common viral pathogens for coinfection were HRSV, HAdV, and HRV, which were also the major respiratory infection pathogens in the pediatric population in China (28). The prevalence of HPIV2 might coincide with these viruses, leading to the frequent occurrence of coinfections, which were also observed in previous studies (29, 30).

The phylogenetic tree revealed that the spread of HPIV2 had spatial clustering. For instance, the sublineage IB2 has been detected only in China thus far, and Chinese sublineage IIE1 was clustered into an independent evolutionary lineage away from the virus from other countries; generally, viruses from the same region/countries tend to cluster together. This phenomenon could also be found in evolution studies of HPIV1 and HPIV3 and might be associated with adaptive epidemics of the virus in the populations of the region/countries (16, 17, 31, 32). Furthermore, the sublineages IB1 and IIE1 have a ladder-like topology, which might reflect immune pressure and rapid viral population expansion (33 to 36). In this study, population expansion of sublineages IB1 and IIE1 during 2010 to 2015 might contribute to shaping this specific topology, but this needs to be confirmed by more genetic data.

The overall evolution rate of HPIV2 based on the HN gene was relatively low, on the order of 10^−4^ substitutions/site/year, while it varied among lineages, with the highest rate of 2.18 × 10^−3^ for sublineage IB2. The rate of HPIV2 was similar to that of the other HPIVs (15, 16, 20, 37, 38). The tMRCA of HPIV2 could be dated to approximately 1922, later than the 1896 predicted by another study due to the differences in the enrolled number of sequences, genes, and sampling time (37). During transmission, HPIV2 formed two distinct clusters in 1934 and 1985 successively, presenting different evolutionary directions of HPIV2. A previous study showed that HPIV3 originated from the common ancestor of bovine parainfluenza virus 3 approximately 200 years ago (38). Accordingly, it was speculated that HPIV2 might have jumped from animal reservoirs to humans approximately 100 years ago. Interestingly, one HPIV2 strain from China in 2019 (strain ZJ-MO3: OM451137) belonging to sublineage IIE1 was isolated from the Malayan pangolin, indicating human–animal transmission of HPIV2. Therefore, close attention to the transmission of HPIV2 between humans and animals is essential (39).

Overall, HPIV2 presented very limited variation at the amino acid level of the HN gene. Selection pressure analysis revealed that the entire HN protein was under purifying/negative selection, indicating a relative conservation of viral evolution (12, 13, 40). Although the nucleotide substitutions were distributed throughout all HN genes, the amino acid changes in sublineages IB1, IB2, and IIE1 were mostly located in the globular head region of the HN protein. Five amino acid substitutions (K316N, K323E, K341N, A416S, and S513N) and two novel N-glycosylation sites (N316 and N517) were shared by the three predominant sublineages. The globular head structural region of the HN protein mediates neuraminidase activity, sialic acid receptor binding, and receptor cleavage, whereas N-glycosylation plays an important role in receptor recognition activity, cleavage of the F protein, and fusion promotion activity in HPIVs (12, 13, 41). The specific amino acid substitutions and addition of glycosylation sites in this region might contribute to the spread of sublineages IB1, IB2, and IIE1.

Although preliminary data for the molecular evolution of HPIV2 were obtained, there are some limitations to the data in this study. (i) Geographical limitations: few HN sequences of HPIV2 have been obtained from the African, Eastern Mediterranean, and Southeast Asian regions so far due to surveillance gaps. (ii) Limitations of chronological distribution: most HN sequences of HPIV2 have only been available in recent years. (iii) Sampling limitation: 68.4% of Chinese HN sequences in this study were from lower respiratory tract infections, which might not include infection cases with mild clinical symptoms. In addition, sequences from elderly or immunocompromised patients were not included. These limitations might lead to deviations in the analytical results of this study.

In summary, the preliminary evolutionary characteristics of HPIV2 based on the HN gene were obtained in this study, thereby increasing the recognition of the evolution and adaptation of HPIV2. However, limited genetic data means that the key mutations for the transmissibility and virulence of HPIV2 are unclear. These results provide a scientific basis for the prevention and control of HPIV2-associated diseases, highlighting the necessity of establishing a surveillance system in China.

## MATERIALS AND METHODS

### Ethical statement and sample collection.

Pharyngeal swab samples were collected from outpatient and inpatient cases diagnosed with ARI in seven provinces of China (Henan, Jilin, Gansu, Chongqing, Shandong, Anhui, and Hebei) in 2011 and 2017 to 2021. Written informed consent for the use of their samples was obtained from all patients or their guardians. Then, the samples were shipped to the National Institute of Viral Disease Control and Prevention, Chinese Center for Disease Control and Prevention and stored at −80°C until use. The common viral pathogens for respiratory infections were screened using the Diagnostic Kit for Respiratory Virus Nucleic Acid (Beijing Kinghawk Pharmaceutical Co., Ltd.).

### HPIV2 detection and sequencing of the HN gene.

Viral RNA was directly extracted from the clinical samples using a commercial nucleic acid extraction kit according to the manufacturer’s instructions (Tianlong Biotechnology Co., Ltd., China, CAT: ZTLJB-Y64). The coding sequence of the HN gene (1,716 nucleotides [nt]) from the HPIV2-positive samples was amplified by reverse transcription PCR (RT-PCR) using two in-house paired primers (Table S1) with the PrimeScript One-Step RT-PCR kit (TaKaRa, Dalian, China). The PCR products were purified using a QIAquick PCR purification kit (Qiagen, Hilden, Germany) and sequenced using an ABI 3130 genetic analyzer (Applied Biosystems, Foster City, CA, USA). The sequencing results were assembled to obtain complete HN sequences using Sequencher v5.4.5 software (GeneCode, Ann Arbor, MI, USA).

### Data set.

In total, 673 available HN sequences of HPIV2 were downloaded from the GenBank database. Incomplete HN gene sequences, sequences with unknown geographic information, sequences with consistent strain names, and identical sequences from the same geographical location at the same collection time were excluded from this study. Finally, a data set containing 90 complete HN sequences covering 15 countries during 1955 to 2019 from GenBank and 21 sequences covering 7 provinces of China in this study was constructed for further analysis. All 111 representative HN sequences of HPIV2 were named with GenBank accession numbers, viruses, countries, strain name, and collection years (Table S2).

### Phylogenetic analysis.

All the 111 sequences were aligned using MAFFT v7.490 software. A phylogenetic tree was constructed based on the HN gene of HPIV2 using the Neighbor-Joining (NJ) algorithm in MEGA v7.0 software. Phylogenetic inference was tested by the bootstrap method with 1,000 replicates (42). The NJ tree was visualized using online software (https://itol.embl.de). The genetic distance (p-distance), sequence identity, and amino acid substitution sites were calculated with MEGA and BioEdit v7.0.9.0 software, respectively.

For the molecular evolution analysis, nine HN sequences from GenBank had been excluded due to the lack of accurate specimen collection date (Table S2). Then, 102 HN gene sequences in the data set were analyzed to infer the evolutionary rate and timescale of HPIV2 using a molecular clock. First, the best-fit model of nucleotide substitution was estimated using JmodelTest 2 v2.1.6 software (Table S3), and based on this result, IQtree v1.6.12 software was used to generate maximum likelihood (ML) phylogenetic trees with 1,000 bootstrap value (43 to 45). Then, the ML tree was used to test the temporal signal of the data set, and a regression of the root-to-tip genetic distances against the year of sampling was generated in TempEst v1.5.3 software (Fig. S1) (46). The results showed that there was clock-like evolution in the data set with a significant regression (*r *= 0.7391).

A Bayesian phylogenetic tree was further constructed based on the HN gene of HPIV2 using the Bayesian Markov chain Monte Carlo (MCMC) algorithm in BEAST v1.10.4 software. After comparing a relaxed molecular clock model and strict molecular clock model with different tree prior probabilities to perform path sampling (PS) and stepping stone sampling (SS) analysis, the convergence of the data set was tested using Tracer v1.7.2 software. Effective sample size (ESS) of >200 was judged as the valid model convergence result. The optimal parameters for the Bayesian phylogenetic model were selected based on the results of the marginal likelihood estimates, which were supported by the PS and SS values (Table S4). According to the results of PS and SS, the suitable molecular clock model was selected for MCMC analysis for different data sets. MCMC chains were run for 200 million steps with sampling every 10,000 generations. The maximum clade credibility (MCC) tree was analyzed using TreeAnnotator v1.10.4 software with 10% burn-in. Figtree v1.4.3 software was used to visualize the MCC tree. The molecular evolution rate and tMRCA of HPIV2 were estimated. The 95% HPD distribution was used to describe the 95% confidence intervals of the analysis results. Additionally, based on PS and SS results, the effective population size of HPIV2 and its main prevalent groups were analyzed by constructing BSP curves, and plots were generated and visualized using Tracer v1.7.2 software (Table S4) (47).

### Natural selection and N-glycosylation site analysis.

Evolutionary pressure values (ω = dN/dS) were calculated using the FEL, MEME, SLAC, and FUBAR algorithms in online software (http://www.datamonkey.org/), where dN denotes the nonsynonymous substitution rate and dS denotes the synonymous substitution rate. For the FEL, MEME, and SLAC algorithms, ω > 1 is a positive selection site, ω = 1 is a neutral selection site, and ω < 1 is a negative selection site when the significant difference is *P *< 0.05. For the FUBAR algorithm, when the posterior probability is >0.95, this indicates the presence of significant positive selection.

The N-glycosylation sites of the HPV2 HN protein were predicted using NetNGlyc v1.0 online software under default parameters (http://www.cbs.dtu.dk/services/NetNGlyc/), suggesting sites with amino acid sequences of Asn-X-Ser/Thr (X is any amino acid except proline) as potential N-glycosylation sites.

### Accession numbers.

Twenty-one HN gene sequences of HPIV2 in this study were submitted to GenBank under accession numbers OP672245 to OP672265 (Table S5).
