# Supplementary material for: Molecular Evolution of Human Parainfluenza Virus Type 2 Based on Hemagglutinin-Neuraminidase Gene
Source: Microbiol Spectr. 2023 Apr 11;11(3):e04537-22. doi: 10.1128/spectrum.04537-22 (PMC10269610; doi:10.1128/spectrum.04537-22)
Supplement: Supplemental file 1 — Supplemental material. Download spectrum.04537-22-s0001.pdf, PDF file, 0.7 MB [file spectrum.04537-22-s0001.pdf]

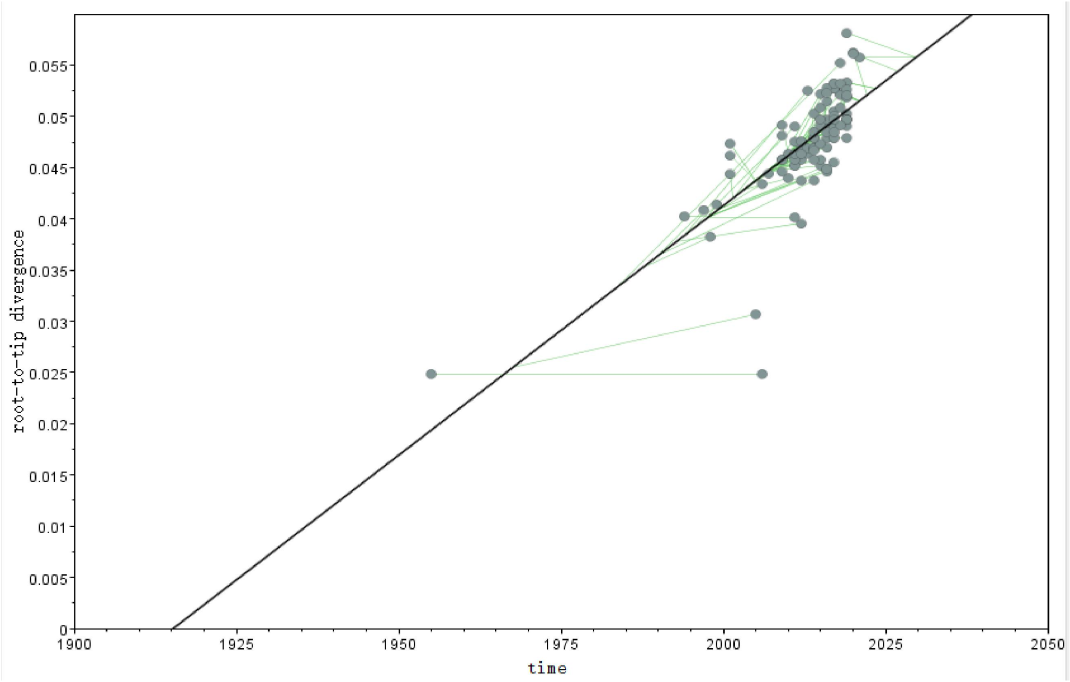

Supplemental Figure 1. Root-to-tip regression of sampling information corresponding to the global datasets for HPIV2.

Supplemental Table 1. The primers for HN gene amplification of HPIV2

Fragment	Primer	Sequence (5' to 3')	Position*	Length (bp)
1	HPIV2-HN+(forward)	CCTGATTATTATGTGATAGCGTAG	6590-6613	2081
	HPIV2-HN-(reverse)	AAGTCGGTGTTGAAGGATTG	8652-8671	
2	HPIV2-HNn+(forward)	AAAATAAGCACGAACCCT	6634-6651	1986
	HPIV2-HNn-(reverse)	CATTCTAATACAGCCAAAAC	8601-8620	

*: referred to prototype strain of HPIV2 (strain GREER: AF533012).

Supplemental Table 2. The information of representative HPIV2 strains in this study

GenBank Number	Country	Strain name	Collection year	WHO Region	Lineage/sublineage	Previous genotype
AF533012	United States	GREER	1955	Americas Region	IA	G4b
JF416793	Korea	KPIV-06-25	2006	Western Pacific Region	IA	G4b
NC_003443	Japan	Toshiba	before 1980	Western Pacific Region	IA	G4b
D00865	United Kingdom	London Colindale	before 1990	European Region	IA	G4b
AB176531	Japan	/	before 2005	Western Pacific Region	IA	G4b
AB367954	Japan	Greer-J1	before 2007	Western Pacific Region	IA	G4b
AB189948	Japan	62-M786	before 1980	Western Pacific Region	outlier	G4a
JF416794	Korea	KPIV-06-26	2006	Western Pacific Region	IB1	G3
MH006623	Vietnam	8	2009	Western Pacific Region	IB1	G3
HM460888	Saudi Arabia	Riyadh_105	2009	Eastern Mediterranean Region	IB1	G3
JF912194	United States	Oklahoma 94	2009	Americas Region	IB1	G3
KT898921	Italy	Pavia VR9944	2009	European Region	IB1	G3
MH006624	Vietnam	14	2010	Western Pacific Region	IB1	G3
MH006625	Vietnam	15	2010	Western Pacific Region	IB1	G3
MG836422	Croatia	Zagreb 17034	2011	European Region	IB1	G3
KT315655	Croatia	Zagreb 16406	2011	European Region	IB1	G3
KT315656	Croatia	Zagreb 16475	2011	European Region	IB1	G3
KT315658	Croatia	Zagreb 16971	2011	European Region	IB1	G3
KM190939	Thailand	VIROAF10	2011	South-East Asia Region	IB1	G3
MG836423	Croatia	Zagreb 2858	2012	European Region	IB1	G3
KT315661	Croatia	Zagreb 2858	2012	European Region	IB1	G3
KT315664	Croatia	Zagreb 3263	2012	European Region	IB1	G3
KT315665	Croatia	Zagreb 3316	2012	European Region	IB1	G3

KT595232	Croatia	Zagreb 2725	2012	European Region	IB1	G3
KX912746	Malaysia	MY-U3258	2013	Western Pacific Region	IB1	G3
KX912743	Malaysia	MY-U2569	2013	Western Pacific Region	IB1	G3
MG836425	Croatia	Zagreb 1472	2014	European Region	IB1	G3
KT315666	Croatia	Zagreb 1472	2014	European Region	IB1	G3
MH892405	Netherlands	t146a292	2014	European Region	IB1	G3
MH828711	Vietnam	vzhpiv22	2014	Western Pacific Region	IB1	G3
MH828712	Vietnam	vzhpiv23	2015	Western Pacific Region	IB1	G3
KY986644	United States	Seattle 10R6	2015	Americas Region	IB1	G3
KY674946	United States	Seattle 9A5	2016	Americas Region	IB1	G3
MK167029	United States	Los_Angeles CHLA26	2016	Americas Region	IB1	G3
MG460775	Croatia	Zagreb 2188	2016	European Region	IB1	G3
MG460784	Croatia	Zagreb 228	2017	European Region	IB1	G3
OP672252	China	HN194	2017	Western Pacific Region	IB1	G3
MT118675	United States	2018_9783	2018	Americas Region	IB1	G3
OP672255	China	GS396	2011	Western Pacific Region	IB2	-
MW575644	China	WH17030d1	2017	Western Pacific Region	IB2	-
OP672251	China	HN173	2017	Western Pacific Region	IB2	-
OP672253	China	HN211	2017	Western Pacific Region	IB2	-
OP672254	China	HN043	2018	Western Pacific Region	IB2	-
OP672245	China	CC105	2019	Western Pacific Region	IB2	-
OP672247	China	CC229	2019	Western Pacific Region	IB2	-
OP672248	China	CC444	2019	Western Pacific Region	IB2	-
OP672246	China	CC-2-001	2020	Western Pacific Region	IB2	-
OP672250	China	AH383	2020	Western Pacific Region	IB2	-
OP672249	China	CQ52	2021	Western Pacific Region	IB2	-

AF533011	United States	V98	1998	Americas Region	outlier	G3
JF912196	United States	Oklahoma 3955	2005	Americas Region	IIC	G2
AB189951	Japan	4-80	before 2004	Western Pacific Region	IIC	G2
AB189952	Japan	TC-6482	before 2004	Western Pacific Region	IIC	G2
OP672256	China	GS191	2011	Western Pacific Region	IID	G1c
KX912738	Malaysia	MY-U1641	2012	Western Pacific Region	IID	G1c
KX912737	Malaysia	MY-U1390	2012	Western Pacific Region	IID	G1c
KX912747	Malaysia	MY-U3500	2014	Western Pacific Region	IID	G1c
MF077311	United States	ACRI_0185	2016	Americas Region	IID	G1c
MW575645	China	WH17080d3	2017	Western Pacific Region	IID	G1c
AF533010	United States	V94	1994	Americas Region	IIE1	G1a
DQ072589	France	Lyon-26056	1997	European Region	IIE1	G1a
AF213352	United States	V9412-6	1999	Americas Region	IIE1	G1a
DQ072586	France	LYON-18620	2001	European Region	IIE1	G1a
DQ072587	France	Lyon-20283	2001	European Region	IIE1	G1a
DQ072588	France	Lyon-20435	2001	European Region	IIE1	G1a
KT898922	Italy	Pavia VR9324	2007	European Region	IIE1	G1a
JF912195	United States	Oklahoma 283	2009	Americas Region	IIE1	G1a
KX912739	Malaysia	MY-U1953	2013	Western Pacific Region	IIE1	G1a
MW654465	Germany	705 14-15	2014	European Region	IIE1	G1a
MW654464	Germany	438 14-15	2014	European Region	IIE1	G1a
KT315667	Croatia	Zagreb 1688	2014	European Region	IIE1	G1a
MH892406	Netherlands	t146a293	2014	European Region	IIE1	G1a
MH828710	Vietnam	vzhpiv21	2014	Western Pacific Region	IIE1	G1a
KY986646	United States	Seattle SC3002	2015	Americas Region	IIE1	G1a
KY986645	United States	Seattle SC2761	2015	Americas Region	IIE1	G1a

MG836418	Croatia	Zagreb 1849	2015	European Region	IIE1	G1a
MF163151	Croatia	Zagreb 1849	2015	European Region	IIE1	G1a
MF163150	Croatia	Zagreb 1779	2015	European Region	IIE1	G1a
MG836417	Croatia	Zagreb 1779	2015	European Region	IIE1	G1a
KY674972	United States	Seattle 14R3	2016	Americas Region	IIE1	G1a
MG836420	Croatia	Zagreb 2513	2016	European Region	IIE1	G1a
MK167022	United States	Los_Angeles CHLA40	2016	Americas Region	IIE1	G1a
MW654466	Germany	271 16-17	2016	European Region	IIE1	G1a
LC654458	United States	NIID_56606_2_2019	2016	Americas Region	IIE1	G1a
MG460772	Croatia	Zagreb 2085	2016	European Region	IIE1	G1a
KY674948	United States	Seattle 9C3	2016	Americas Region	IIE1	G1a
MW654472	Germany	4782 16-17	2017	European Region	IIE1	G1a
MG460785	Croatia	Zagreb 705	2017	European Region	IIE1	G1a
MG773274	Argentina	BuenosAires 1	2017	Americas Region	IIE1	G1a
MF077313	United States	ACRI_0248	2017	Americas Region	IIE1	G1a
MW654471	Germany	1668 16-17	2017	European Region	IIE1	G1a
MW654473	Germany	1398 17-18	2017	European Region	IIE1	G1a
MF077312	United States	ACRI_0230	2017	Americas Region	IIE1	G1a
OP672265	China	HN185	2017	Western Pacific Region	IIE1	G1a
MN369034	United States	Seattle SC9949	2018	Americas Region	IIE1	G1a
MW654475	Germany	566 18-19	2018	European Region	IIE1	G1a
MW654477	Germany	737 18-19	2018	European Region	IIE1	G1a
OP672257	China	HN405	2019	Western Pacific Region	IIE1	G1a
OP672258	China	HN428	2019	Western Pacific Region	IIE1	G1a
OP672259	China	QD279	2019	Western Pacific Region	IIE1	G1a
OP672260	China	CC091	2019	Western Pacific Region	IIE1	G1a

OP672261	China	HB20	2019	Western Pacific Region	IIE1	G1a
OP672262	China	CC338	2019	Western Pacific Region	IIE1	G1a
OP672263	China	HN389	2019	Western Pacific Region	IIE1	G1a
OP672264	China	HN491	2019	Western Pacific Region	IIE1	G1a
MT118716	United States	2019_2885	2019	Americas Region	IIE1	G1a
MW654479	Germany	1637 18-19	2019	European Region	IIE1	G1a
OM451137	China	ZJ-MO3	2019	Western Pacific Region	IIE1	G1a
MW654476	Germany	712 18-19	2018	European Region	outlier	-
AB189949	Japan	86-391	before 2004	Western Pacific Region	IIE2	G1b
AB189950	Japan	76-86	before 2004	Western Pacific Region	IIE2	G1b

Note： The grey area presented the sequence from mainland China in 2011 and 2017-2021 in this study. "/" indicated the sequence without strain name. "-" indicated the sequences without genotyping information.

Supplemental Table 3. Best-fit model of nucleotide substitution selected for MCMC analysis for different datasets

Dataset	Nucleotide substitution model
ALL HPIV2	GTR+G
Cluster I	GTR+G
Cluster II	GTR+I
IB	GTR+G
IB1	HKY+I
IB2	HKY
IIE	HKY+I
IIE1	HKY+I

Supplemental Table 4. The results of path sampling and stepping stone sampling analysis for different datasets

Dataset	Molecular clock model	Coalescent tree prior	ESS	PS	SS
HPIV2-ALL	Strick clock	GMRF Bayesian Skyride	＞200	-7677.200	-7676.922
	Strick clock	Constant Size	＞200	-7680.214	-7680.510
	Strick clock	Exponential Growth	＞200	-7678.849	-7678.948
	Strick clock	Bayesian Skyline	＞200	-7682.653	-7683.116
	Uncorrelated exponential relaxed clock	GMRF Bayesian Skyride	＞200	-7811.531	-7813.203
	Uncorrelated exponential relaxed clock	Constant Size	＞200	-7797.659	-7789.822
	Uncorrelated exponential relaxed clock	Exponential Growth	＞200	-7801.883	-7798.611
	Uncorrelated exponential relaxed clock	Bayesian Skyline	＞200	-7800.317	-7796.051
	Uncorrelated lognormal relaxed clock	GMRF Bayesian Skyride	＞200	-7815.736	-7805.680
	Uncorrelated lognormal relaxed clock	Constant Size	＞200	-7798.292	-7789.421
	Uncorrelated lognormal relaxed clock	Exponential Growth	＞200	-7795.941	-7789.876
	Uncorrelated lognormal relaxed clock	Bayesian Skyline	＞200	-7797.787	-7793.444
HPIV2-Cluster Ⅰ	Strick clock	GMRF Bayesian Skyride	＞200	-4517.006	-4513.936
	Strick clock	Constant Size	＞200	-4511.896	-4508.861
	Strick clock	Exponential Growth	＞200	-4511.488	-4508.820
	Strick clock	Bayesian Skyline	＞200	-4517.545	-4515.520
	Uncorrelated exponential relaxed clock	GMRF Bayesian Skyride	＞200	-4503.747	-4501.721
	Uncorrelated exponential relaxed clock	Constant Size	＞200	-4503.288	-4500.449
	Uncorrelated exponential relaxed clock	Exponential Growth	＞200	-4504.838	-4502.444
	Uncorrelated exponential relaxed clock	Bayesian Skyline	＞200	-4504.553	-4502.473
	Uncorrelated lognormal relaxed clock	GMRF Bayesian Skyride	＞200	-4512.017	-4508.711
	Uncorrelated lognormal relaxed clock	Constant Size	＞200	-4508.698	-4506.089
	Uncorrelated lognormal relaxed clock	Exponential Growth	＞200	-4509.893	-4506.521
	Uncorrelated lognormal relaxed clock	Bayesian Skyline	＞200	-4510.965	-4507.326
HPIV2-Cluster Ⅱ	Strick clock	GMRF Bayesian Skyride	＞200	-5519.300	-5515.811
	Strick clock	Constant Size	＞200	-5523.857	-5522.190
	Strick clock	Exponential Growth	＞200	-5521.513	-5518.197
	Strick clock	Bayesian Skyline	＞200	-5523.117	-5520.196
	Uncorrelated exponential relaxed clock	GMRF Bayesian Skyride	＞200	-5536.897	-5533.066
	Uncorrelated exponential relaxed clock	Constant Size	＞200	-5539.668	-5537.292
	Uncorrelated exponential relaxed clock	Exponential Growth	＞200	-5538.674	-5536.062
	Uncorrelated exponential relaxed clock	Bayesian Skyline	＞200	-5539.723	-5536.779
	Uncorrelated lognormal relaxed clock	GMRF Bayesian Skyride	＞200	-5527.243	-5524.489
	Uncorrelated lognormal relaxed clock	Constant Size	＞200	-5532.119	-5532.491
	Uncorrelated lognormal relaxed clock	Exponential Growth	＞200	-5533.116	-5528.708
	Uncorrelated lognormal relaxed clock	Bayesian Skyline	＞200	-5528.557	-5526.010

HPIV2-IB	Strick clock	GMRF Bayesian Skyride	＞200	-3765.589	-3763.954
	Strick clock	Constant Size	＞200	-3765.864	-3764.261
	Strick clock	Exponential Growth	＞200	-3765.407	-3763.785
	Strick clock	Bayesian Skyline	＞200	-3764.656	-3762.891
	Uncorrelated exponential relaxed clock	GMRF Bayesian Skyride	＞200	-3773.115	-3772.254
	Uncorrelated exponential relaxed clock	Constant Size	＞200	-3771.451	-3769.790
	Uncorrelated exponential relaxed clock	Exponential Growth	＞200	-3773.168	-3771.519
	Uncorrelated exponential relaxed clock	Bayesian Skyline	＞200	-3769.916	-3769.212
	Uncorrelated lognormal relaxed clock	GMRF Bayesian Skyride	＞200	-3771.419	-3769.769
	Uncorrelated lognormal relaxed clock	Constant Size	＞200	-3774.079	-3773.831
	Uncorrelated lognormal relaxed clock	Exponential Growth	＞200	-3775.036	-3770.884
	Uncorrelated lognormal relaxed clock	Bayesian Skyline	＞200	-3773.183	-3772.904
HPIV2-IB1	Strick clock	GMRF Bayesian Skyride	＞200	-3336.870	-3336.294
	Strick clock	Constant Size	＞200	-3338.777	-3337.903
	Strick clock	Exponential Growth	＞200	-3334.424	-3334.290
	Strick clock	Bayesian Skyline	＞200	-3333.344	-3332.564
	Uncorrelated exponential relaxed clock	GMRF Bayesian Skyride	＞200	-3339.924	-3338.917
	Uncorrelated exponential relaxed clock	Constant Size	＞200	-3346.456	-3345.100
	Uncorrelated exponential relaxed clock	Exponential Growth	＞200	-3337.171	-3336.424
	Uncorrelated exponential relaxed clock	Bayesian Skyline	＞200	-3340.015	-3340.639
	Uncorrelated lognormal relaxed clock	GMRF Bayesian Skyride	＞200	-3343.962	-3342.860
	Uncorrelated lognormal relaxed clock	Constant Size	＞200	-3347.029	-3345.821
	Uncorrelated lognormal relaxed clock	Exponential Growth	＞200	-3343.073	-3343.081
	Uncorrelated lognormal relaxed clock	Bayesian Skyline	＞200	-3342.088	-3341.319
HPIV2-IB2	Strick clock	GMRF Bayesian Skyride	＜200	-3008.007	-3007.326
	Strick clock	Constant Size	＜200	-3007.619	-3006.749
	Strick clock	Exponential Growth	＞200	-3009.457	-3009.039
	Strick clock	Bayesian Skyline	＞200	-3007.021	-3006.370
	Uncorrelated exponential relaxed clock	GMRF Bayesian Skyride	＜200	-3005.498	-3005.205
	Uncorrelated exponential relaxed clock	Constant Size	＜200	-3005.453	-3004.774
	Uncorrelated exponential relaxed clock	Exponential Growth	＞200	-3005.813	-3005.287
	Uncorrelated exponential relaxed clock	Bayesian Skyline	＜200	-3011.187	-3011.807
	Uncorrelated lognormal relaxed clock	GMRF Bayesian Skyride	＞200	-3008.446	-3007.711
	Uncorrelated lognormal relaxed clock	Constant Size	＞200	-3008.557	-3007.627
	Uncorrelated lognormal relaxed clock	Exponential Growth	＜200	-3008.131	-3007.569
	Uncorrelated lognormal relaxed clock	Bayesian Skyline	＞200	-3009.285	-3008.197
HPIV2-IIE	Strick clock	GMRF Bayesian Skyride	＞200	-4834.784	-4833.828
	Strick clock	Constant Size	＞200	-4837.221	-4834.765
	Strick clock	Exponential Growth	＞200	-4835.660	-4833.477
	Strick clock	Bayesian Skyline	＞200	-4835.114	-4834.445
	Uncorrelated exponential relaxed clock	GMRF Bayesian Skyride	＞200	-4847.534	-4845.947
	Uncorrelated exponential relaxed clock	Constant Size	＞200	-4847.738	-4846.403

	Uncorrelated exponential relaxed clock	Exponential Growth	＞200	-4845.744	-4845.094
	Uncorrelated exponential relaxed clock	Bayesian Skyline	＞200	-4853.498	-4851.791
	Uncorrelated lognormal relaxed clock	GMRF Bayesian Skyride	＞200	-4841.752	-4839.127
	Uncorrelated lognormal relaxed clock	Constant Size	＞200	-4843.402	-4840.173
	Uncorrelated lognormal relaxed clock	Exponential Growth	＞200	-4843.840	-4840.874
	Uncorrelated lognormal relaxed clock	Bayesian Skyline	＞200	-4842.326	-4839.368
HPIV2-IIE1	Strick clock	GMRF Bayesian Skyride	＞200	-4486.482	-4484.909
	Strick clock	Constant Size	＞200	-4490.594	-4487.873
	Strick clock	Exponential Growth	＞200	-4490.380	-4491.531
	Strick clock	Bayesian Skyline	＞200	-4486.115	-4483.047
	Uncorrelated exponential relaxed clock	GMRF Bayesian Skyride	＞200	-4495.577	-4494.503
	Uncorrelated exponential relaxed clock	Constant Size	＞200	-4493.699	-4491.076
	Uncorrelated exponential relaxed clock	Exponential Growth	＞200	-4492.927	-4490.999
	Uncorrelated exponential relaxed clock	Bayesian Skyline	＞200	-4496.027	-4494.093
	Uncorrelated lognormal relaxed clock	GMRF Bayesian Skyride	＞200	-4494.530	-4492.791
	Uncorrelated lognormal relaxed clock	Constant Size	＞200	-4494.690	-4492.148
	Uncorrelated lognormal relaxed clock	Exponential Growth	＞200	-4493.294	-4490.996
	Uncorrelated lognormal relaxed clock	Bayesian Skyline	＞200	-4493.653	-4491.551

Note： Light blue area indicated the best molecular clock model and the tree priors. The red font referred to the parametric model used to construct Bayesian skyline plot.

Supplemental Table 5. The clinical information of HPIV2 infection cases in this study

Strain	Gender	Age(year)	Date of Onset	Collection date	Detection result [a]	Clinical diagnosis[b]	Sample type [c]	GenBank accession No.
GS191	Male	1	2011/2/21	2011/2/21	HPIV2	URTI	P	OP672256
GS396	Male	3	2011/9/17	2011/9/17	HPIV2	URTI	P	OP672255
HN173	Female	1	2017/10/10	2017/10/17	HPIV2/HRSV	B	P	OP672251
HN185	Male	5	2017/10/22	2017/10/26	HPIV2	AB	P	OP672265
HN194	Male	4	2017/10/25	2017/10/28	HPIV2	-	P	OP672252
HN211	Male	6	2017/10/27	2017/11/2	HPIV2/EV	B	P	OP672253
HN043	Female	2	2018/1/8	2018/1/13	HPIV2	-	P	OP672254
HN389	Male	4	2019/7/16	2019/7/20	HPIV2/HRV/229E/HUK1/HAdV	B	P	OP672263
HN405	Male	2	2019/8/10	2019/8/13	HPIV2/HRV/HAdV	AL	P	OP672257
HN428	Male	3	2019/9/23	2019/9/26	HPIV2	AL	P	OP672258
HN491	Female	4	2019/11/16	2019/11/26	HPIV2/OC43	AB	P	OP672264
CC091	Male	9	2019/7/15	2019/7/18	HPIV2/FLUB	AB、AL	P	OP672260
CC105	Female	2	2019/7/15	2019/7/19	HPIV2	B	P	OP672245
CC229	Female	4	2019/9/21	2019/9/28	HPIV2	AB	P	OP672247
CC338	Female	2	2019/12/21	2019/12/24	HPIV2	B、AL	P	OP672262
CC444	Male	1	2019/12/29	2020/1/1	HPIV2	AURTI	P	OP672248
QD279	Male	1	2019/9/26	2019/9/26	HPIV2	P	N/P	OP672259
HB20	Male	3	2019/8/6	2019/8/6	HPIV2 /HAdV	P	P	OP672261
CC-2-001	Male	5	2020/4/20	2020/4/27	HPIV2	P	P	OP672246
AH383	Male	4	2020/12/6	2020/12/8	HPIV2/HRSV	URTI	N/P	OP672250
CQ-52	Male	12	2021/1/21	2021/1/21	HPIV2/HRV/EV/HRSV	B、BA	N	OP672249

Note:

a: HPIV2: Human Parainfluenza virus type 2; HRSV: Human respiratory syncytial virus; EV: Enterovirus; HRV: Human rhinovirus; 229E: Human coronavirus 229E; HUK1: Human coronavirus HKU1; HAdV: Human adenovirus; OC43: Human coronavirus OC43; FLUB: Influenza B virus;

b: URTI: upper respiratory tract infection; AURTI: acute upper respiratory tract infection; B: bronchopneumonia; AB: acute bronchitis; AL: acute laryngitis; P: pneumonia; BA: bronchial asthma.

c: P: pharyngeal swabs; N: nasal swabs；

"-" indicated the cases without clinical diagnosis information.

Supplemental Table 6. Genetic distance between and within lineages of HPIV2

lineage	IA	62-M786*	IB	IIC	IID	IIE
IA	**0.003**					
62-M786*	0.019	-				
IB	0.038	0.036	**0.014**			
IIC	0.049	0.051	0.069	**0.007**		
IID	0.059	0.060	0.077	0.020	**0.011**	
IIE	0.061	0.064	0.078	0.025	0.031	**0.013**

Note: Bold numbers were genetic distances within the lineages of HPIV2.

*: Strain 62-M786 (AB189948) referred to the outlier in this study.

Supplemental Table 7. Genetic distance between and within lineage IB of HPIV2

Sublineage	IB1	IB2	V98*
IB1	**0.006**		
IB2	0.026	**0.008**	
V98*	0.014	0.021	-

Note: Bold numbers were genetic distances within the sublineage IB1 and sublineage IB2 of HPIV2.

*: Strain V98 (AF533011) referred to the outlier in this study.

Supplemental Table 8. P-distance among HPIV2 lineage IIE in this study

Sublineage	IIE1	0712*	IIE2
IIE1	**0.012**		
0712*	0.034	-	
IIE2	0.020	0.026	**0.006**

Note: Bold numbers were genetic distances within the sublineage IIE1 and sublineage IIE2 of HPIV2.

*: Strain 0712 (MW654476) referred to the outlier in this study.

Supplemental Table 9. The summary of representative HPIV2 strains used in this study

Cluster	Lineage /Sub-lineage	Previous genotype	Number of Sequences	Period	Country	WHO Region
I	IA	G4b	6	1955-2006	JPN UK KOR USA	Americas/European/Western Pacific
	IB	G3	43	1998-2021	CHN USA KOR VNM SAU HRV THA MYS NLD ITA	Americas/European/Western Pacific/South-East Asia/Eastern Mediterranean
	IB1	G3	31	2009-2018	CHN USA KOR VNM SAU HRV THA MYS NLD ITA	Americas/European/Western Pacific/South-East Asia/Eastern Mediterranean
	IB2	-	11	2011-2021	CHN	Western Pacific
II	IIC	G2	3	before2004-2005	JPN USA	Americas/Western Pacific
	IID	G1c	6	2011-2017	CHN USA MYS	Americas/Western Pacific
	IIE	G1a、G1b	52	1994-2019	CHN GER USA HRV ARG NLD VNM ITA MYS FRA JPN	Americas/European/Western Pacific
	IIE1	G1a	49	1994-2019	CHN GER USA HRV ARG NLD VNM ITA MYS FRA	Americas/European/Western Pacific
	IIE2	G1b	2	-	JPN	Western Pacific

Note: The country abbreviation ARG, CHN, FRA, GER, HRV, ITA, JPN, KOR, MYS, NLD, SAU, THA, UK, USA and VNM in the trees presented Argentina, China, France, Germany, Croatia, Italy, Japan, Korea, Malaysia, the Netherlands, Saudi Arabia, Thailand, the United

Kingdom, United States and Vietnam, respectively. "-" indicates the lineage/sublineage without previous genotype or circulation period information.

Supplemental Table 10. Amino acid substitution of lineage IB compared to the HPIV2 prototype strain

Lineage/Subineage	Stalk			Head															
	54	67	100	164	175	316	319	323	332	341	345	351	360	367	376	416	497	513	514
GREER	D	I	Y	N	I	K	P	K	K	K	Q	S	N	V	H	A	R	S	A
IB	N	V	-	H	S	N	-	E	-	N	-	G	-	I	Q	S	K	N	S
IB1	N	V	-	H	S	N	-	E	-	N	-	G	Y	I	Q	S	K	N	S
IB2	N	V	L	H	S	N	S	E	T	N	R	G	-	I	Q	S	K	N	S

Note: "-" indicated the amino acid sites without substitution.

Supplemental Table 11. Amino acid substitution of lineage IIC compared to the HPIV2 prototype strain

Lineage	Stalk			Head																	
	57	100	114	139	195	201	211	316	319	323	341	344	348	379	416	479	482	497	513	514	571
GREER	D	Y	T	K	T	A	A	K	P	K	K	E	A	R	A	P	Q	R	S	A	P
IIC	E	L	A	E	A	S	E	N	T	E	N	K	I	G	S	L	R	K	N	S	L

Supplemental Table 12. Amino acid substitution of lineage IID compared to the HPIV2 prototype strain

Lineage	Stalk			Head																			
	57	100	114	139	195	201	211	254	281	316	319	323	341	348	379	416	479	480	497	512	513	514	571
GREER	D	Y	T	K	T	A	A	E	S	K	P	K	K	A	R	A	P	T	R	A	S	A	P
IID	E	L	A	E	A	S	G	D	F	N	T	E	N	I	E	S	L	M	K	T	N	S	L

Supplemental Table 13. Amino acid substitution of lineage IIE compared to the HPIV2 prototype strain

Lineage/ Subineage	Stalk			Head																					
	57	100	114	139	195	201	211	316	319	323	341	344	348	378	379	402	416	479	480	482	497	513	514	570	571
GREER	D	Y	T	K	T	A	A	K	P	K	K	E	A	A	R	D	A	P	T	Q	R	S	A	I	P
IIE	-	-	-	E	A	S	-	-	T	E	N	-	I	E	-	-	S	-	M	R	-	N	-	M	L
IIE1	-	L	-	E	A	S	-	N	T	E	N	-	I	E	E	-	S	-	M	R	-	N	-	M	L
IIE2	E	-	A	E	A	S	G	-	T	E	N	K	I	E	-	G	S	L	M	R	K	N	S	M	L

Note: "-" indicated the amino acid sites without substitution.

Supplemental Table 14. Amino acid substitution of lineage IB compared to earliest reported strain of lineage IB

Sublineage	Stalk		Head							
	48	117	139	254	276	322	348	360	381	381
V98	A	T	K	E	V	N	A	N	D	D
IB1	-	-	-	-	-	-	-	Y	-	-
IB2	V	-	-	-	-	-	-	-	-	-
> 20%*	V	S	N	D	I	K	T	Y	N	S

Note: "-" indicated the amino acid sites without substitution.

*: those amino acid substitutions observed in more than 20% of sequences.

Supplemental Table 15. Amino acid substitution of lineage IIE compared to earliest reported strain of lineage IIE

Subineage	Stalk			Head					
	54	57	87	137	164	186	254	351	476
V94	N	E	V	V	H	M	E	S	D
IIE2	D	-	I	-	N	I	-	-	-
> 20%*	-	D	-	A	-	I	D	G	N

Note: "-" indicated the amino acid sites without substitution.

*: those amino acid substitutions observed in more than 20% of sequences.

Supplemental Table 16. N-glycosylation site prediction based on HPIV2 HN protein

Lineage/sublineage	GenBank Number	N-glycosylation site					
		6	115	142	272	316	517
IA	AF533012	N	N	N	N	-	-
IA	JF416793	N	N	N	N	-	-
IA	NC_003443	N	N	N	N	-	-
IA	D00865	N	N	N	N	-	-
IA	AB176531	N	N	N	N	-	-
IA	AB367954	N	N	N	N	-	-
IB1	JF416794	N	N	N	N	N	N
IB1	MH006623	N	N	N	N	N	N
IB1	HM460888	N	N	N	N	N	N
IB1	JF912194	N	N	N	N	N	N
IB1	KT898921	N	N	N	N	N	N
IB1	MH006624	N	N	N	N	N	N
IB1	MH006625	N	N	N	N	N	N
IB1	MG836422	N	N	N	N	N	N
IB1	KT315655	N	N	N	N	N	N
IB1	KT315656	N	N	N	N	N	N
IB1	KT315658	N	N	N	N	N	N
IB1	KM190939	N	N	N	N	N	N
IB1	MG836423	N	N	N	N	N	N
IB1	KT315661	N	N	N	N	N	N
IB1	KT315664	N	N	N	N	N	N
IB1	KT315665	N	N	N	N	N	N
IB1	KT595232	N	N	N	N	N	N
IB1	KX912746	N	N	N	N	N	N
IB1	KX912743	N	N	N	N	N	N
IB1	MG836425	N	N	N	N	N	N
IB1	KT315666	N	N	N	N	N	N
IB1	MH892405	N	N	N	N	N	N
IB1	MH828711	N	N	N	N	N	N
IB1	MH828712	N	N	N	N	N	N
IB1	KY986644	N	N	N	N	N	N
IB1	KY674946	N	N	N	N	N	N
IB1	MK167029	N	N	N	N	N	N
IB1	MG460775	N	N	N	N	N	N
IB1	MG460784	N	N	N	N	N	N
IB1	OP672252	N	N	N	N	N	N

IB1	MT118675	N	N	N	N	N	N
IB2	OP672255	N	N	N	N	N	N
IB2	MW575644	N	N	N	N	N	N
IB2	OP672251	N	N	N	N	N	N
IB2	OP672253	N	N	N	N	N	N
IB2	OP672254	N	N	N	N	N	N
IB2	OP672245	N	N	N	N	N	N
IB2	OP672247	N	N	N	N	N	N
IB2	OP672248	N	N	N	N	N	N
IB2	OP672246	N	N	N	N	N	N
IB2	OP672250	N	N	N	N	N	N
IB2	OP672249	N	N	N	N	N	N
IIC	JF912196	N	N	N	N	N	N
IIC	AB189951	N	N	N	N	N	N
IIC	AB189952	N	N	N	N	N	N
IID	OP672256	N	N	N	N	N	N
IID	KX912738	N	N	N	N	N	N
IID	KX912737	N	N	N	N	N	N
IID	KX912747	N	N	N	N	N	-
IID	MF077311	N	N	N	N	N	N
IID	MW575645	N	N	N	N	N	N
IIE1	AF533010	N	N	N	N	N	N
IIE1	DQ072589	N	N	N	N	N	N
IIE1	AF213352	N	N	N	N	N	N
IIE1	DQ072586	N	N	N	N	N	N
IIE1	DQ072587	N	N	N	N	N	N
IIE1	DQ072588	N	N	N	N	N	N
IIE1	KT898922	N	N	N	N	N	N
IIE1	JF912195	N	N	N	N	N	N
IIE1	KX912739	N	N	N	N	N	N
IIE1	MW654465	N	N	N	N	N	N
IIE1	MW654464	N	N	N	N	N	N
IIE1	KT315667	N	N	N	N	N	N
IIE1	MH892406	N	N	N	N	N	N
IIE1	MH828710	N	N	N	N	N	N
IIE1	KY986646	N	N	N	N	N	N
IIE1	KY986645	N	N	N	N	N	N
IIE1	MG836418	N	N	N	N	N	N
IIE1	MF163151	N	N	N	N	N	N
IIE1	MF163150	N	N	N	N	N	N
IIE1	MG836417	N	N	N	N	N	N

IIE1	KY674972	N	N	N	N	N	N
IIE1	MG836420	N	N	N	N	N	N
IIE1	MK167022	N	N	N	N	-	N
IIE1	MW654466	N	N	N	N	N	N
IIE1	LC654458	N	N	N	N	N	N
IIE1	MG460772	N	N	N	N	N	N
IIE1	KY674948	N	N	N	N	N	N
IIE1	MW654472	N	N	N	N	N	N
IIE1	MG460785	N	N	N	N	N	N
IIE1	MG773274	N	N	N	N	N	N
IIE1	MF077313	N	N	N	N	N	N
IIE1	MW654471	N	N	N	N	N	N
IIE1	MW654473	N	N	N	N	N	N
IIE1	MF077312	N	N	N	N	N	N
IIE1	OP672265	N	N	N	N	N	N
IIE1	MN369034	N	N	N	N	N	N
IIE1	MW654475	N	N	N	N	N	N
IIE1	MW654477	N	N	N	N	N	N
IIE1	OP672257	N	N	N	N	N	N
IIE1	OP672258	N	N	N	N	N	N
IIE1	OP672259	N	N	N	N	N	N
IIE1	OP672260	N	N	N	N	N	N
IIE1	OP672261	N	N	N	N	N	N
IIE1	OP672262	N	N	N	N	N	N
IIE1	OP672263	N	N	N	N	N	N
IIE1	OP672264	N	N	N	N	N	N
IIE1	MT118716	N	N	N	N	N	N
IIE1	MW654479	N	N	N	N	N	-
IIE1	OM451137	N	N	N	N	N	N
IIE2	AB189949	N	-	N	N	-	N
IIE2	AB189950	N	N	N	N	N	N
outlier (62-M786)	AB189948	N	N	-	N	-	N
outlier (V98)	AF533011	N	N	N	N	N	N
outlier (712 18-19)	MW654476	N	N	N	N	N	N

Note: "-" indicated the sites without predicted N-glycosylation.

Supplemental Table 17. Negative selection sites estimated by four algorithms for sublineage IB1

Codon	SLAC	SLAC ω	FEL	FEL ω	MEME	MEME ω	FUBAR
96	0.071	< 1	**0.025**	< 1	0.67	< 1	**0.006**
97	**0.037**	< 1	**0.012**	< 1	0.67	< 1	**0.001**
109	0.112	< 1	**0.036**	< 1	0.67	< 1	**0.010**
123	0.346	< 1	0.175	< 1	0.67	< 1	**0.025**
191	0.111	< 1	**0.045**	< 1	0.67	< 1	**0.010**
210	**0.037**	< 1	**0.010**	< 1	0.67	< 1	**0.000**
229	0.06	< 1	**0.014**	< 1	0.67	< 1	**0.001**
362	0.283	< 1	0.085	< 1	0.67	< 1	**0.015**
418	0.125	< 1	0.053	< 1	0.67	< 1	**0.005**
467	0.111	< 1	0.051	< 1	0.67	< 1	**0.010**
502	0.244	< 1	**0.034**	< 1	0.67	< 1	**0.006**
531	**0.037**	< 1	**0.026**	< 1	0.67	< 1	**0.001**

Note: Bold numbers indicated that pressure selection site was supported by the algorithm.

Supplemental Table 18. Negative selection sites estimated by four algorithms for sublineage IB2

Codon	SLAC	SLAC ω	FEL	FEL ω	MEME	MEME ω	FUBAR
14	0.211	< 1	**0.047**	< 1	0.067	< 1	0.051
123	0.317	< 1	0.01	< 1	0.067	< 1	**0.013**
188	0.349	< 1	**0.047**	< 1	0.067	< 1	0.053
204	0.214	< 1	**0.036**	< 1	0.067	< 1	0.051

Note: Bold numbers indicated that pressure selection site was supported by the algorithm.

Supplemental Table 19. Negative selection sites estimated by four algorithms for lineage IID

Codon	SLAC	SLAC ω	FEL	FEL ω	MEME	MEME ω	FUBAR
348	0.118	< 1	**0.014**	< 1	0.67	< 1	0.067

Note: Bold numbers indicated that pressure selection site was supported by the algorithm.

Supplemental Table 20. Negative selection sites estimated by four algorithms for sublineage IIE1

Codon	SLAC	SLAC ω	FEL	FEL ω	MEME	MEME ω	FUBAR
62	0.07	< 1	**0.018**	< 1	0.067	< 1	**0.013**
91	**0.019**	< 1	**0.002**	< 1	0.067	< 1	**0.000**
93	0.079	< 1	**0.017**	< 1	0.067	< 1	**0.019**
104	**0.019**	< 1	**0.002**	< 1	0.067	< 1	**0.000**
116	0.086	< 1	**0.023**	< 1	0.067	< 1	**0.019**
144	**0.037**	< 1	**0.022**	< 1	0.067	< 1	**0.002**
163	0.111	< 1	**0.042**	< 1	0.067	< 1	**0.024**
194	**0.037**	< 1	**0.020**	< 1	0.067	< 1	**0.002**
209	0.111	< 1	**0.015**	< 1	0.067	< 1	**0.014**
240	0.111	< 1	**0.019**	< 1	0.067	< 1	**0.021**
243	0.337	< 1	0.114	< 1	0.067	< 1	**0.031**
258	0.111	< 1	0.062	< 1	0.067	< 1	**0.023**
306	0.102	< 1	**0.043**	< 1	0.067	< 1	**0.026**
331	0.111	< 1	**0.022**	< 1	0.067	< 1	**0.011**
334	0.111	< 1	**0.042**	< 1	0.067	< 1	**0.024**

362	**0.045**	< 1	**0.005**	< 1	0.067	< 1	**0.000**
388	**0.037**	< 1	**0.019**	< 1	0.067	< 1	**0.003**
419	0.111	< 1	**0.049**	< 1	0.067	< 1	**0.026**
421	0.088	< 1	**0.043**	< 1	0.067	< 1	**0.022**
443	0.089	< 1	**0.019**	< 1	0.067	< 1	**0.012**
500	0.111	< 1	0.058	< 1	0.067	< 1	**0.027**
502	0.266	< 1	**0.048**	< 1	0.067	< 1	**0.016**
566	0.184	< 1	**0.034**	< 1	0.067	< 1	**0.002**
567	0.112	< 1	**0.035**	< 1	0.067	< 1	**0.021**

Note: Bold numbers indicated that pressure selection site was supported by the algorithm.

Supplemental Table 21. Positive selection sites estimated by four algorithms for lineage IID and sublineage IB1, IB2, IIE1

lineage/ sublineage	Codon	SLAC	SLAC ω	FEL	FEL ω	MEME	MEME ω	FUBAR
IID	515	0.444	> 1	0.15	> 1	0.07	=1	**0.953**
IB1	479	0.296	> 1	0.09	> 1	0.11	=1	**0.972**
IB2	350	0.315	> 1	0.101	> 1	**0.05**	=1	0.938
IIE1	87	0.218	> 1	0.144	> 1	0.17	=1	**0.954**
IIE1	351	0.251	> 1	0.202	> 1	0.22	=1	**0.959**

Note: Bold numbers indicated that pressure selection site was supported by the algorithm.
